# Effects of Prokineticins on Cerebral Cell Function and Blood–Brain Barrier Permeability

**DOI:** 10.3390/ijms242015428

**Published:** 2023-10-21

**Authors:** Hadi Younes, Ioanna Kyritsi, Zineb Mahrougui, Mohamed Benharouga, Nadia Alfaidy, Christel Marquette

**Affiliations:** 1 University Grenoble-Alpes, CEDEX 9, 38043 Grenoble, France; hadiyounes25@gmail.com (H.Y.); ioanna_kir@hotmail.com (I.K.); zinebmahrougui@hotmail.com (Z.M.); mohamed.benharouga@cea.fr (M.B.); nadia.alfaidy-benharouga@cea.fr (N.A.); 2 Commissariat à l’Energie Atomique et aux Energies Alternatives (CEA), Laboratory of Biology & Biotechnology for Health, Interdisciplinary Research Institute of Grenoble, 38000 Grenoble, France; 3 Institut National de la Santé et de la Recherche Médicale U1292, Biologie et Biotechnologie pour la Santé, 38000 Grenoble, France

**Keywords:** prokineticin, prokineticin receptor, receptors antagonists, blood–brain barrier integrity, brain endothelial cells

## Abstract

Prokineticins are a family of small proteins with diverse roles in various tissues, including the brain. However, their specific effects on different cerebral cell types and blood–brain barrier (BBB) function remain unclear. The aim of this study was to investigate the effects of PROK1 and PROK2 on murine cerebral cell lines, bEnd.3, C8.D30, and N2a, corresponding to microvascular endothelial cells, astrocytes and neurons, respectively, and on an established BBB co-culture model. Western blot analysis showed that prokineticin receptors (PROKR1 and PROKR2) were differentially expressed in the considered cell lines. The effect of PROK1 and PROK2 on cell proliferation and migration were assessed using time-lapse microscopy. PROK1 decreased neural cells’ proliferation, while it had no effect on the proliferation of endothelial cells and astrocytes. In contrast, PROK2 reduced the proliferation of all cell lines tested. Both PROK1 and PROK2 increased the migration of all cell lines. Blocking PROKRs with the PROKR1 antagonist (PC7) and the PROKR2 antagonist (PKR-A) inhibited astrocyte PROK2-mediated migration. Using the insert co-culture model of BBB, we demonstrated that PROKs increased BBB permeability, which could be prevented by PROKRs’ antagonists.

## 1. Introduction

Prokineticins are small proteins (8 kDa) that bind to GPCRs and play critical roles in several biological functions. They are involved in the stimulation of smooth muscle contraction and can induce proliferation, migration, and fenestration of endothelial cells (EC) [1,2,3,4,5,6]. Prokineticin 1 (PROK1) is predominantly expressed by endocrine glands in humans, while prokineticin 2 (PROK2) is more commonly found outside of these glands [5]. Prokineticin receptor 1 (PROKR1), the type one receptor of PROK1 and PROK2, is mainly found in peripheral tissues, while prokineticin receptor 2 (PROKR2) is more highly expressed in the brain, contributing to the complexity of this system [7]. Prokineticins demonstrate significant potential to promote cell survival and proliferation in different cell types, including endothelial cells [8], neural cells [9], lymphocytes, hematopoietic stem cells, and cardiomyocytes [10]. They activate signaling pathways such as MAPK, AKT, and PI3K to enhance cell survival and regulate endothelial cell behavior [9,11,12,13]. 

PROK1 acts specifically on endothelial cells and mediates angiogenesis through PROKR1. It stimulates EC cell proliferation and migration and contributes to vessel branching [14,15,16,17].

In the heart and kidneys, activation of PROKR2 in endothelial cells results in vascular fenestration and increased permeability, either by increasing the formation of vesiculo-vesicular permeability organelles and caveolae, and/or by altering tight junction assembly and downregulating ZO-1-mediated cell–cell adhesion [18]. 

In the central and peripheral nervous systems, PROK2 is more expressed than PROK1, and displays several physiological functions. PROK2 acts as an output signal of the circadian clock, synchronizing the endogenous behavioral circadian rhythms [19]. PROK2 acts as a neurotrophic factor, promoting neural survival [20,21], while PROK1 stimulates the proliferation of neural precursors in the enteric nervous system [12]. PROKR2 is widely expressed in various brain areas (olfactory bulb, striatum, hippocampus, thalamus hypothalamus, suprachiasmatic nucleus, amygdala, and neocortex), while PROKR1 has only been detected in the neocortex [22]. PROK2 and PROKR2 proteins are highly expressed in adult neurogenic zones. It has been shown that the chemotactic property of PROK2 allows for the migration of neural precursors to the olfactory bulb (OB) and regulates its morphogenesis [23]. Thus, the PROK2/PROKR2 axis in the OB plays a role in the development and migration/survival of GnRH neurons [23,24,25], and mutations in PROK2 and PROK2 genes lead to a defect in OB neuron migration associated with Kallmann syndrome [26]. The induction of PROK2 expression in response to pathological insults, such as ischemia and stroke, contributes to inflammation and negatively affects the pathophysiological response. Inhibition of PROK2 signaling using receptor antagonists reduces inflammation and the infarct volume of ischemia, suggesting that it may serve as a potential therapeutic approach to mitigate the detrimental effects of ischemic stroke. [27]. However, at high concentrations, PROK2 may play a crucial neuroprotective role during cerebral injury through the activation of the ERK1/2 and AKT pathways [28]. In the context of traumatic brain injury, PROK2 has been reported to contribute to cell recruitment within injured areas, hence preventing neural cell death [23,29,30]. 

The effect of PROKs on the functions of the cerebral endothelium, known as the blood–brain barrier (BBB), has not been specifically studied and remains to be investigated. The vasculature of the central nervous system (CNS) possesses a set of properties to form a barrier that controls the movement of ions, nutrients, and cells between the blood and the CNS. The neurovascular unit (NVU) of the BBB is composed of endothelial cells that line the blood vessels of the brain; a basement membrane that lies beneath the endothelial cells and provides structural support; pericytes that wrap around the endothelial cells; and astrocytes’ endfeet that surround the blood vessels. The endothelial cells of the BBB differ from those of the peripheral organs in several features, including a lack of fenestrations, reduced transcytosis, expression of tight junctions, and lower permeability [31,32,33,34,35]. Taken together, these processes contribute to the maintenance of vascular integrity, which warrants normal cerebral functions. Dysfunction of the BBB is one of the main causes of the symptoms of dementia, as they occur in neurological and neurodegenerative diseases. Disruption of the BBB during cerebral ischemia results in nutrient deficiency, edema formation, and excitotoxic cell death [36,37]. In the ischemic nuclease, hypoxia and glutamate release activate various genes that produce proteins with both beneficial and deleterious effects. This cascade of events exacerbates inflammation which activates endothelial cells and macrophages, and recruits T cells through the affected BBB. This process ultimately leads to tissue damage [38]. 

The aim of this study was to investigate the effect of the PROKs/PROKRs system on the proliferation and migration of cerebral cells, in particular microvascular endothelial cells, astrocytes, and neurons, and to evaluate the influence of prokineticin on the permeability of the BBB in a murine in vitro BBB model, using a contact co-culture system of endothelial cells in association with astrocytes.

## 2. Results

### 2.1. Expression of Prokineticin Receptors in Cerebral Cells 

The study of the impact of prokineticins on the BBB is crucial due to the BBB’s role as the first point of contact with the circulating blood within the brain. Therefore, our first experiments were conducted to determine the levels of expression of prokineticin receptors in the cells constituting the neurovascular unit, the NVU. Specifically, we focused on three murine cell lines derived from the brain: bEnd.3 cells as an endothelial cell line, the C8-D30 cells (type III clone) cells as an astrocyte cell line, and N2a murine as a neuroblastoma cell line. 

**Figure 1:**
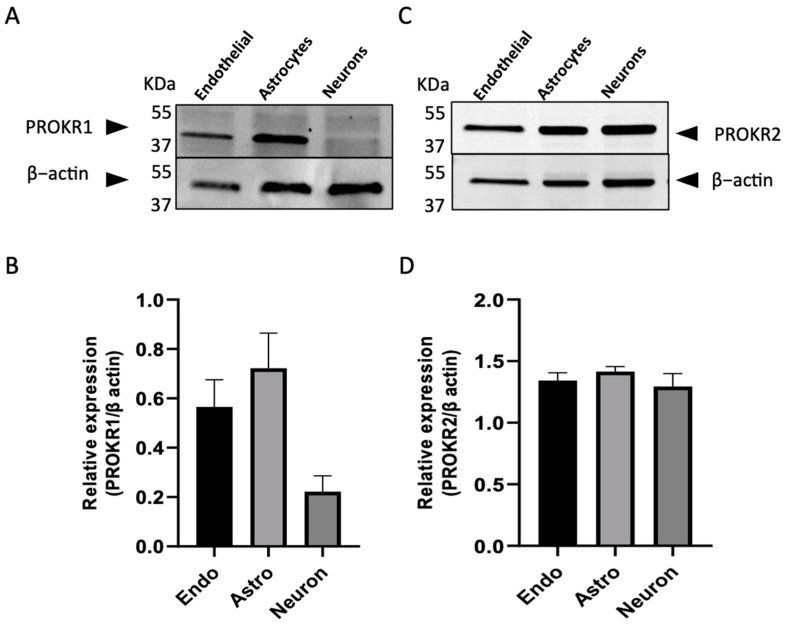
Expression of PROKR1 and PROKR2 proteins in b.End.3, C8-D30, and N2a cell lines. (**A**) Representative Western blot analysis of PROKR1 expression in b.End.3 (endothelial cells), C8-D30 (astrocytes), and N2a (neurons) cells. (**B**) Quantification of PROKR1 expression levels. (**C**) Representative Western blot analysis of PROKR2 expression in the three cell lines, b.End.3, C8-D30, and N2a. (**D**) Quantification of PROKR2 expression levels. Quantification was performed with Image-J (3.53). Data are presented as mean ± SEM, from three independent experiments.

Figure 1A,B show that PROKR1 protein detected at a molecular weight (MW) of 45 KDa was expressed both in endothelial cells and astrocytes, while its expression in N2a cells was negligible. On the other hand, PROKR2 was detected at 44 KDa in all cell types. The quantification of the corresponding bands indicated relatively similar levels of expression across the different cell types (Figure 1C,D). Western blot analyses indicate that the examined cell lines, especially endothelial and astrocytes, express both PROKRs. Hence, these cell lines can serve as valuable tools for assessing the effects of the ligands PROK1 and PROK2 in the model of the blood–brain interface established with bEnd.3 and C8D30 cells.

### 2.2. Effect of PROKs on the Proliferation of Cerebral Cells 

PROKs have been characterized as angiogenic and mitogenic factors in various cell types. 

Investigating PROKs’ effects on proliferation and migration, especially on cerebrovascular endothelial cells, astrocytes and neurons, is of the utmost importance for a better understanding of the complex cellular processes underlying brain neurodevelopment, its vascular homeostasis, and associated neurological disorders. 

The experimental procedure consisted of seeding bEnd.3, C8-D30, and N2a cells (6 × 10^3^ cells/well) in a 96-well plate and allowing them to adhere for one day. Different concentrations of PROK1 or PROK2, ranging from 0.5 to 30 nM, were then used as treatments.

**Figure 2:**
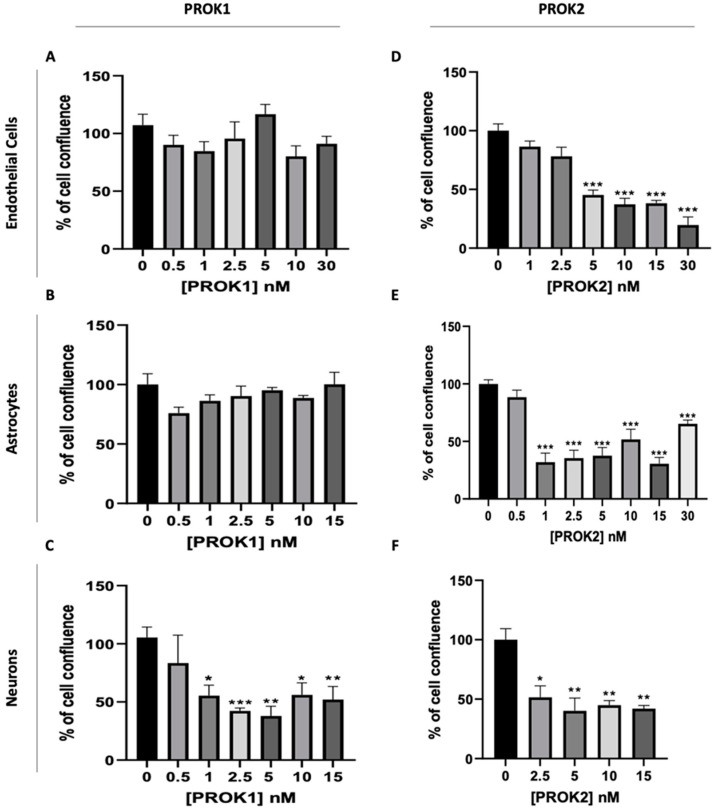
Effect of PROK1 and PROK2 on the proliferation of b.End3, C8-D30, and N2a cell lines. Effect of PROK1 on the proliferation of (**A**) endothelial cells (b.End.3), (**B**) astrocytes (C8-D30), and (**C**) neurons (N2a). Effect of PROK2 on the proliferation of (**D**) endothelial cells, (**E**) astrocytes, and (**F**) neurons. Data are presented as mean ± SEM, from three independent experiments; unpaired two-tailed *t* test was used for statistical analysis and statistical significance is indicated as * *p* ≤ 0.05, ** *p* ≤ 0.01, *** *p* ≤ 0.001, compared to the condition without prokineticin.

The results depicted in Figure 2A,B demonstrated that the treatment of endothelial cells and astrocytes with PROK1 did not have any significant effect on their proliferation. However, when neurons were treated with PROK1, a significant decrease in their proliferation was observed with a dose of 1 nM (Figure 2C). In contrast, treatment with PROK2 resulted in a significant decrease in the proliferation of all cell types investigated. Notably, endothelial cells’ proliferation was significantly reduced at the concentration of 5 nM of PROK2 and this effect increased with increasing doses (Figure 2D). Conversely, both astrocytes and neurons exhibited a significant decrease in their proliferation from 1 nM and 2.5 nM of PROK2, respectively (Figure 2E,F).

### 2.3. Effect of PROKs on the Migration of Cerebral Cells

Investigation of the effects of PROKs on cell migration was performed on the same murine cell lines (endothelial cells, astrocytes, and neurons). After allowing the cells to reach confluence in a 96-well plate over the course of one day, a wound was made in each well. PROK1 or PROK2 ligands were then added at concentrations ranging from 1 to 15 nM, along with mitomycin to inhibit cell proliferation. This approach allowed us to focus only on cell migration, as proliferation was effectively blocked. 

**Figure 3:**
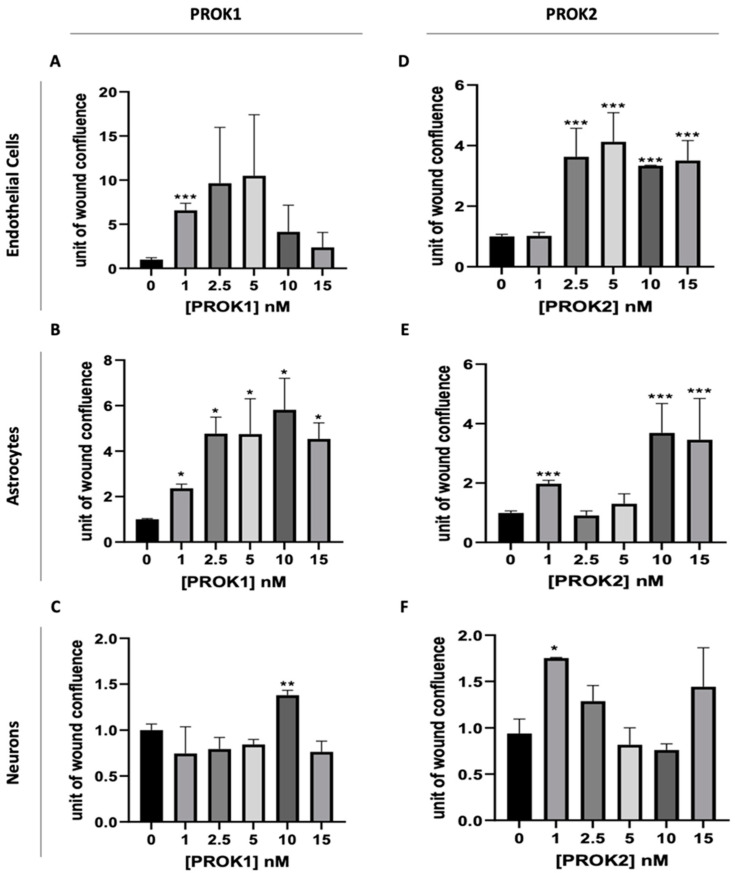
Effect of PROK1 and PROK2 on the migration of b.End.3, C8-D30, and N2a cell lines. Effect of PROK1 on the migration of (**A**) endothelial cells (b.End.3), (**B**) astrocytes (C8-D30), and (**C**) neurons (N2a). Effect of PROK2 on the migration of (**D**) endothelial cells, (**E**) astrocytes, and (**F**) neurons. Data are presented as mean ± SEM, from three independent experiments; unpaired two-tailed *t* test was used for statistical analysis and statistical significance is indicated as * *p* ≤ 0.05, ** *p* ≤ 0.01, *** *p* ≤ 0.001 compared to the condition without prokineticin.

The results, presented in Figure 3, showed that the treatment with either PROK1 or PROK2 induced an increase in the migration of all cell types, albeit at different concentrations.

In response to PROK1, endothelial cells exhibited a significant increase in migration at a concentration of 1 nM (Figure 3A). Astrocytes displayed an increase in cell migration at all concentrations tested (Figure 3B). For neurons, we demonstrated that PROK1 had a significant effect on neural cell migration only at a concentration of 10 nM (Figure 3C). In the case of PROK2, we observed an effect on endothelial cell migration from a concentration of 2.5 nM (Figure 3D). No additional effect on these cells’ migration was observed when this concentration was increased. For astrocytes, PROK2 induced an increase in cell migration at concentrations of 1, 10, and 15 nM, while 2.5 and 5 nM did not exhibit any significant effect (Figure 3E). Finally, for neurons, PROK2 exhibited a slight increase in their migration only at the lowest concentration tested, i.e., 1 nM (Figure 3F).

### 2.4. Effect of PROKRs’ Antagonists on the Migration of Cerebral Cells

To further characterize the mechanism by which PROK2 regulates the migration of astrocytes, we blocked their receptors using PROKR1 and PROKR2’s respective antagonists, PC-7 and PKR-A. These antagonists were added to the astrocytes’ cultures 2 h prior to PROK2 treatment and wound-healing creation.

**Figure 4:**
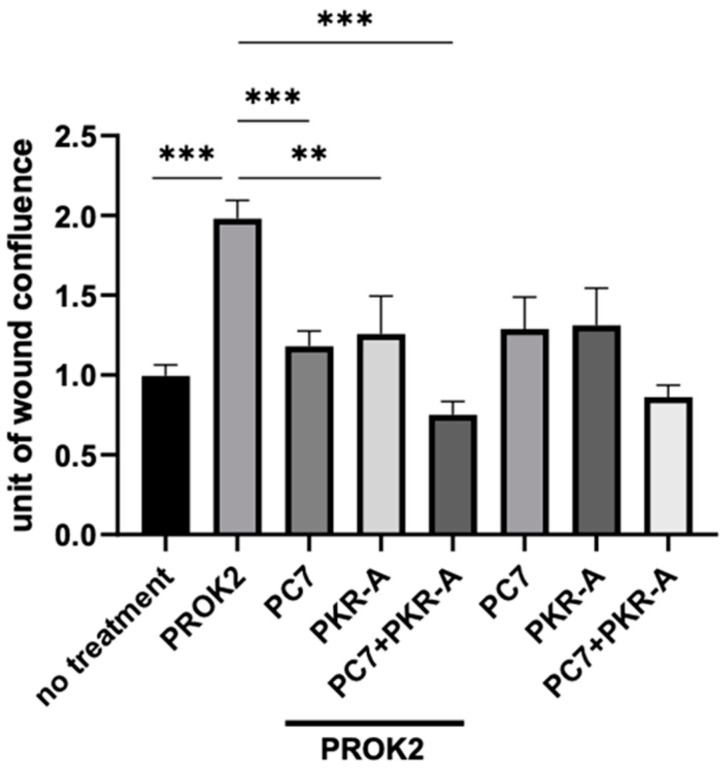
Effect of PROKR1 and PROKR2 antagonists, PC-7 and PKR-A, on astrocyte migration. Data are presented as mean ± SEM, from three independent experiments; ordinary one-way ANOVA, corrected by Sidak test for multiple comparisons, was used for statistical analysis and statistical significance is indicated as ** *p* ≤ 0.01, *** *p* ≤ 0.001.

The results, as shown in Figure 4, demonstrated that the addition of PROKR1 antagonist, PROKR2 antagonist, or a combination of both prior to the treatment with the ligand, efficiently prevented PROK2-induced astrocytes’ migration. However, when the antagonist was added alone in the absence of PROK2, no significant effect on the astrocytes’ migration was observed.

These results strongly suggest that PROK2-mediated astrocyte migration is dependent on the activation of PROKR1 and PROKR2, as their antagonists effectively inhibit the PROK2-mediated migration process.

### 2.5. Effect of PROKs and PROKRs’ Antagonist on BBB Co-Culture Model

To investigate the effect of prokineticins on the BBB, we established an in vitro co-culture model consisting of immortalized murine brain endothelial cells bEnd.3 and murine astrocytes C8-D30 cell lines. The integrity and tightness of this model were assessed by measuring the transendothelial electrical resistance (TEER) values and permeability across the barrier. Subsequently, the effects of prokineticins on this co-culture model were examined in the absence and presence of receptor antagonists.

#### 2.5.1. Assessment of the TEER and Permeability of the Established In Vitro BBB Model

Two strategies were conducted to set up the optimal conditions for the use of the BBB. The first one used a monolayer culture of endothelial cells plated at the apical side of the insert, while the second one consisted of a co-culture of endothelial cells plated on the apical side and astrocytes on the basolateral side of the insert.

**Figure 5:**
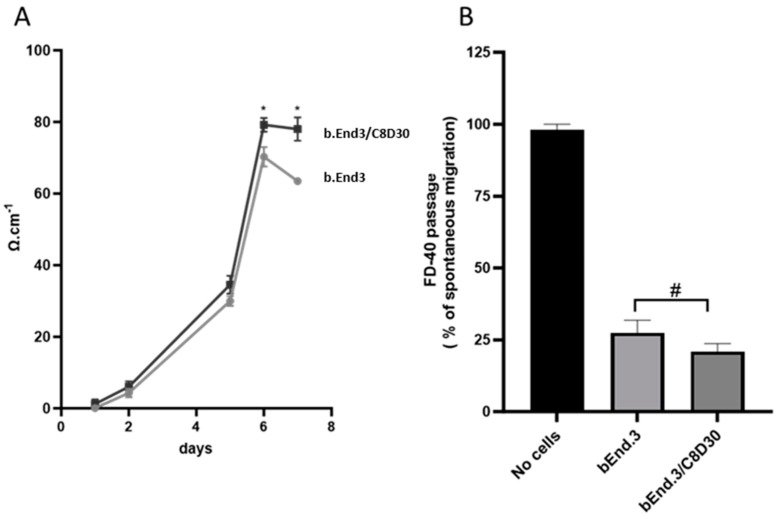
Assessment of transendothelial electrical resistance (TEER) and FD-40 passage in monolayer and co-culture models. (**A**) TEER measurements of endothelial cell monolayer and endothelial with astrocyte co-culture models were recorded over a 7-day culture period, with values presented in ohm.cm^2^. (**B**) The reduction of spontaneous passage of FD-40 across the endothelial monolayer and co-culture models compared to the spontaneous migration of FD-40 across the insert without cells. Data are presented as mean ± SEM, and representative of three independent experiments. Statistical analysis: two-way ANOVA corrected by Sidak test for multiple comparisons was used for TEER measurements and unpaired one-tailed *t*-test was used for FD-40 passage measurements. Statistical significance is indicated as * *p* < 0.05 and # *p* = 0.1.

After 6 days of culture, the TEER values of the inserts with both endothelial cells, on the apical side, and astrocytes, on the basolateral side, reached approximately 80 Ω.cm^2^. These values were higher than those measured in the monolayer culture model, which recorded approximately 75 Ω.cm^2^ (Figure 5A). On the 6th day, TEER values of the co-culture were maintained, reaching plateau values, while TEER values of the monoculture significantly dropped.

The values of the spontaneous passage of Fluorescein isothiocyanate-dextran (FD-40) were significantly reduced by 73% in the case of the b.End3 monolayer culture and by 80% in the case of the co-culture with C8-D30, compared to the control consisting of the insert without cells. Furthermore, a significant difference was observed between the two culture conditions, suggesting that the presence of astrocytes improved the BBB tightness (see Figure 5B). 

Thus, the co-culture of endothelial and astrocyte cells proves to be a stronger model for the forthcoming experiments to assess the impact of PROK1 and PROK2 and their respective receptor antagonists on the permeability of the BBB.

#### 2.5.2. Prokineticins’ Effect on the Integrity of the BBB 

The effects of PROK1 and PROK2 on the integrity of the BBB were examined at day 6–7 of co-culture, when TEER measurements of the co-culture model reached a plateau. The ligands were then added at various concentrations (10–60 nM) and incubated for 24 h. The measurement of the permeability was based on the measurement of FD-40 transfer.

**Figure 6:**
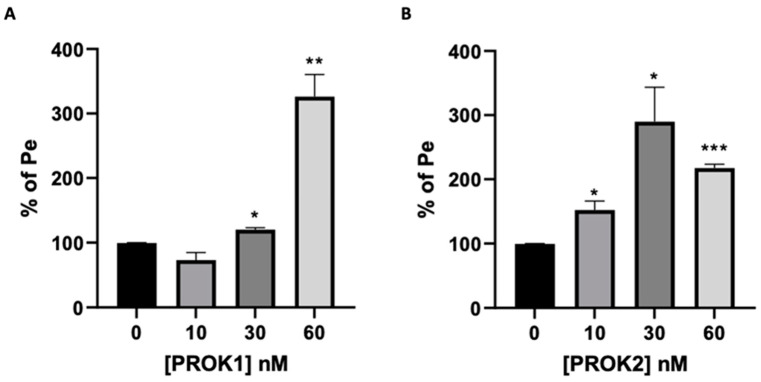
Effect of PROKs on the permeability of the BBB model. Permeability assessment of the co-culture of bEnd.3/C8-D30 cells 24 h after treatment with PROK1 (**A**) or with PROK2 (**B**). Data are expressed as a percentage of permeability coefficient (Pe) (as mean ± SEM) compared to untreated control. Statistical analysis: unpaired two-tailed *t*-test was used from three independent experiments. Statistical significance is indicated as * *p* ≤ 0.05, ** *p* ≤ 0.01, *** *p* ≤ 0.001, compared to the condition without prokineticin.

Treatment with PROK1 resulted in a significant increase in the permeability at concentrations of 30 and 60 nM (Figure 6A). Similarly, treatment with PROK2 also increased the permeability of the co-culture model at the concentrations tested (10 to 60 nM) (Figure 6B). These results demonstrate that both PROK1 and PROK2 alter the BBB integrity by increasing its permeability.

#### 2.5.3. Effect of PROKRs’ Antagonists on the Permeability of the BBB Co-Culture Model

To investigate the direct effect of prokineticins on the integrity of the BBB, two prokineticin receptor antagonists, PC-7 and PKR-A, were added to the BBB co-culture 1 h before ligand treatment, either alone or combined.

**Figure 7:**
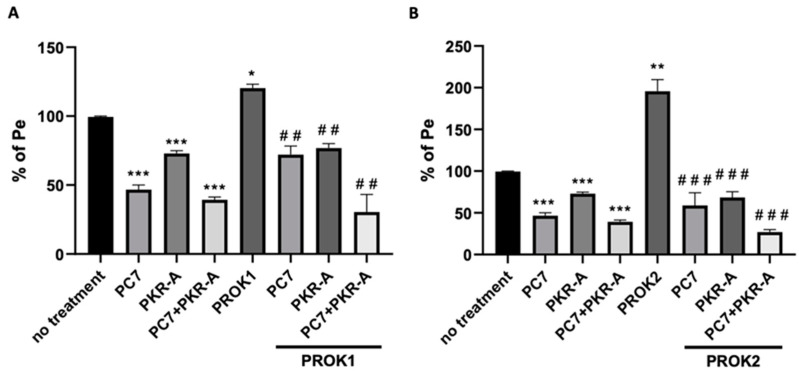
Effect of PROKR antagonists on the permeability of the BBB co-culture model. Measurement of the permeability of the co-culture model was assessed in the presence or absence of PROK1 (30 nM) (**A**) or PROK2 (60 nM) (**B**), pretreated with PROKR antagonists, PC7 or PKR-A (1 µM). Data are presented as a percentage of permeability coefficient (Pe) compared to untreated insert, as mean ± SEM, from three independent experiments. Statistical analysis: unpaired two-tailed *t*-test was used. Statistical significance is indicated as * *p* ≤ 0.05, ** *p* ≤ 0.01, *** *p* ≤ 0.001, compared to CTL, and ^##^ *p* ≤ 0.01, ^###^ *p* ≤ 0.001 compared to PROK1 or PROK2 treatments when used alone.

Treatment of the co-cultures with either PC7 or PKR-A antagonists alone, or combined, resulted in a significant decrease in the permeability of the BBB. These effects were observed in the absence of any treatment with the ligands (Figure 7). In addition, a synergistic effect was observed when both PC7 and PKR-A were used together. PROK1 used at 30 nM significantly increased the permeability of the BBB. The blockade of the receptors with either PC7 or PKR-A, or with a combination of both, prevented the increase in permeability induced by PROK1 (Figure 7A). The maximum inhibitory effect was observed when both receptor antagonists were used. A similar effect was observed for PROK2, when used at 60 nM. The blockade was observed when either antagonist was used or when combined (Figure 7B). These results indicate that PROK1 and PROK2 exert their effects on the permeability of the BBB through both receptors. The blockade of either receptor may prevent their potential adverse effects on the BBB. It can be noted that PC7 was more effective in restricting permeability than PKR-A.

## 3. Discussion

### 3.1. PROK1 and PROK2 Act on Cerebral Cells’ Proliferation and Migration

Accumulating evidence points to alterations in brain vascular function in several neuropathologic conditions such as cerebral edema, epilepsy, Alzheimer’s disease, and Parkinson’s disease [21,22,31,32,39]. Since prokineticins have recently been recognized as potential targets for the treatment of the neuroinflammation observed in stroke/ischemia, neurodegenerative diseases, or dementia related to vascular dysfunction [40], the aim of the present study was to characterize the effects of prokineticin family members on the cerebrovascular interface between blood and brain tissue by studying the BBB in vitro. Indeed, any disruption of the barrier formed by cerebrovascular cells (endothelial cells, pericytes, and astrocytes) may compromise the proper assembly and maintenance of tight junctions, resulting in the increased permeability and infiltration of harmful substances into the brain [32,33,41,42]. Occupying a strategic position between capillaries and neurons, astrocytes, with perivascular endfeet at the BBB, play a prominent role in maintaining ionic, amino acid, neurotransmitter, and water homeostasis in the brain. Moreover, upon insult, neural cells can secrete inflammatory factors, including PROKs, which can modulate the integrity of the BBB [43,44]. 

For endothelial cell proliferation, PROK1 did not significantly affect cell proliferation at any concentration tested (from 0.5 to 30 nM), while PROK2 induced a decrease in the proliferation of bEnd.3 with an IC50 of 3 nM. Previous studies have shown that PROK1 can stimulate the proliferation of primary bovine adrenal cortex-derived capillary endothelial cells with an ED50 of 0.2 nM, with a maximal effect observed at 2 nM [5] of bovine brain capillary endothelial cells at concentrations from 10 to 100 nM [5]. In addition, primary placental microvascular endothelial cells responded positively to PROK1 when used at 2.5 nM [14]. However, no effect of PROK1 on the proliferation of HUVEC, umbilical vein-derived macrovascular cells, was observed at low (1–5 nM) [14] or high (10–100 nM) [5] concentrations. In addition, no effect was observed in either human dermal microvascular cells or adult bovine aortic endothelial cells (10–100 nM) [5]. On the other hand, PROK2 has been reported to increase the proliferation of coronary endothelial cells (H5V) (1–5 nM) [13]. These discrepancies may be related to the cell types of the organ used, as well as to the doses and duration of the treatments. Nevertheless, it can be speculated that the lack of effects of PROK1 on brain endothelial cells’ proliferation may be due to their non-fenestrated nature, which makes them less prone to proliferation and response to angiogenic factors [6]. 

Similarly, PROK2 showed a decrease in the proliferation of astrocytes with an IC50 of 0.52 nM, whereas PROK1 had no significant effect on the astrocyte cell line at all concentrations tested (0.5–15 nM). These results are in opposition to previous studies showing a positive effect of both PROK1 and PROK2 at 10 nM on primary mouse astrocytes [45,46]. Further studies are needed to confirm whether this discrepancy is due to the use of an astrocyte cell line rather than primary astrocyte cultures. A significant decrease in neural proliferation was observed when treated with PROK1 and PROK2 with an IC50 of 0.58 nM and 3.4 nM, respectively. This finding is contradictory to previous studies indicating that PROK1 stimulates the proliferation of neural precursors in the enteric nervous system (mouse embryonic intestinal neural crest cells) and the human neuroblastoma cell line SK-N-SH. Both studies used PROK1 at pharmacological concentrations (200–400 nM) [9,12], suggesting that this ligand may have different effects at physiological and pharmacological/pathological concentrations. 

With respect to brain cell migration, both PROK1 and PROK2 affected the migration of the cell types studied. PROK1 treatment increased the migration of endothelial cells and astrocytes at a low concentration (1 nM), whereas at higher concentrations (10 and 15 nM) this effect was attenuated or even reversed. PROK1 exhibited an effect on neural migration only at the higher concentration of 10 nM. These results are in agreement with previous studies showing the involvement of PROK1 especially in cell migration of endothelial cells HPEC (2.5 nM) [14], ACE (0.5–5 nM), and BAEC (endothelial cells from baboon adrenal cortex) (0.5–5 nM), and the MS-1 cell line (microvessels of mouse endocrine pancreas) (5 nM) [5]. In contrast, PROK1 had no effect on HUVEC cell migration [5,14]. In neurons, PROK1, but not PROK2, enhances cell migration of the human neuroblastoma cell line SK-N-SH at high concentrations (400 nM) [12]. However, at the same concentration, PROK1 did not affect the migration of mouse embryonic intestinal neural crest cells (400 nM) [9]. In the case of PROK2, treatment stimulated endothelial cell and astrocyte migration with an EC50 of 2.3 and 5.6 nM, respectively, while PROK2 appears to stimulate neural migration at low concentrations but has no or the opposite effect at higher concentrations (2.5–15 nM). Others have also shown that PROK2 induces the migration of H5V endothelial cells at 5 nM [13] and primary astrocytes at 25 nM [45]. In our study only the lowest concentration of 10 nM stimulated the migration of neural cell lines, which was described with olfactory bulb neural precursors and the regulation of their morphogenesis [23]. The addition of PROKR antagonists to the C8-D30 cultures prevented PROK2-mediated migration, as reported in primary mouse astrocytes [45], confirming that the migration of astrocytes is dependent on the activation of PROKR1 and PROKR2.

The observed differential response among the same cell types may be due to several factors, such as the origin of the cells (e.g., human, mouse, or other animal types) and their features (primary cells or immortalized cell lines). These factors can lead to variations in the overall expression of PROKs’ receptors, which in turn can affect the functional outcome by altering signaling pathways. Another explanation for the variability in the cells’ responses to PROKs’ ligands may be related to the levels of expression of their receptor at the cell membrane and to their affinity for prokineticins. Although PROKR1 and PROKR2 share 87% homology in their amino acid sequences, and can both bind the two receptors with different affinities, the resulting response may differ depending on the repertoire of G proteins and the level of different subunits present in each cell type. The specific combination of G protein subunits associated with the prokineticin receptors can influence downstream signaling events and ultimately determine the cellular response [47].

### 3.2. Effect of PROKs and PROKRs’ Antagonists on BBB

The observed increase in TEER values and decreased spontaneous passage of FD-40 in the co-culture with bEnd.3 and C8-D30 cell lines, compared to the monolayer endothelial cell culture, strongly suggests that the presence of astrocytes improves BBB tightness. These results are consistent with previous studies using bEnd.3 and C8-D30 cell lines in co-culture, which have also displayed the critical role of astrocytes in maintaining the integrity and function of the BBB [32,48,49,50,51,52]. Thus, this model effectively mimics the integrity and tightness of the BBB, making it suitable for studying the effects of prokineticins and their respective receptor antagonists on its permeability. However, this model has some limitations, as the establishment of barrier conditions is limited to 2 days, although the use of primary cells should allow the barrier to be established over several days [53]. Another limitation of the model is the absence of shear stress, which has been shown to affect barrier tightness and tight junction protein expression [54].

Subsequent experiments with PROK1 and PROK2 showed that both prokineticins significantly increased BBB permeability in a concentration-dependent manner. Further studies with specific PROK receptor antagonists, PC7 and PKR-A, provided valuable insights into the underlying mechanisms of prokineticins on BBB permeability. Blocking either PROKR1 or PROKR2 individually resulted in a significant reduction in BBB permeability, suggesting that both receptors play an important role in mediating the disruptive effects of prokineticins. Notably, the combined inhibition of PROKR1 and PROKR2 showed a synergistic effect, further confirming the involvement of both receptors in the regulation of BBB permeability. The effect of PROKR antagonists alone on endothelial cells’ permeability, without the presence of the ligands, has also been reported in previous studies. The silencing or blocking of PROKRs resulted in reduced sprouting and permeability of HPEC cells [14] and reduced proliferation of the choriocarcinoma cell line JEG3 [55]. This effect may be attributed to the autocrine/paracrine mode of action of the prokineticin ligand, which might be secreted under normal conditions to maintain normal physiological cell function. By antagonizing the receptors, we may be able to prevent the aberrant, non-physiological actions of the prokineticin system [56,57,58,59,60]. 

**Figure 8:**
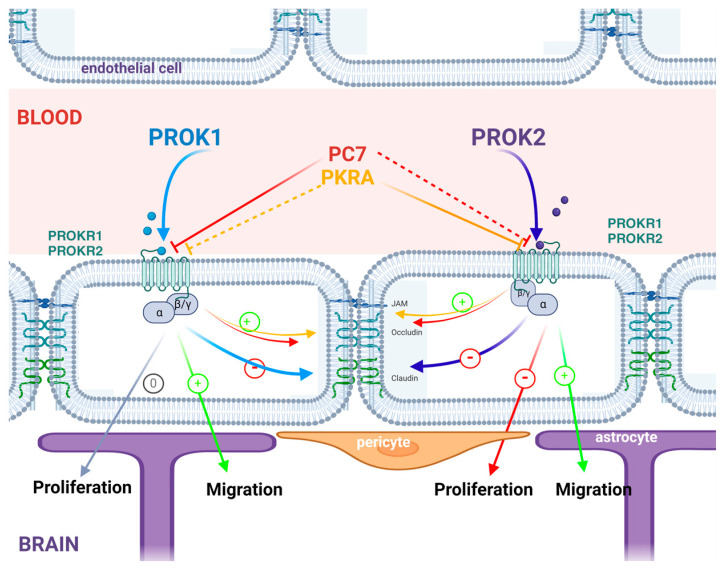
Recapitulative schematic with hypothetical action of PROKs and PROKR antagonists’ effect on the permeability of the BBB co-culture model. The solid line indicates a preferential effect of the antagonist on the prokineticin receptor, compared with a less preferential activity indicated by the dotted line. Created with BioRender.com, https://app.biorender.com/illustrations/651bda235b60aacc08b683d9 (accessed on 5 September 2023).

This study is the first to show that both PROK1 and PROK2 may directly affect the integrity and permeability of the blood–brain barrier. This is consistent with previous studies demonstrating the importance of the PROKs/PROKRs system in the endothelium signaling of numerous tissues [5,6,13,14,61,62,63]. The mechanisms involved remain to be explored, such as the signaling pathway activated by PROKs to control cell permeability. One can speculate that PROKs may activate Gα12 signaling, known to be involved in the modification of tight junction and protein cell–cell interaction (Figure 8), as previously described for VEGF signaling [64]. 

These results may allow for the integration of the PROKs/PROKRs system in physiological and pathological cerebrovascular processes with potential consideration of their targeting in new pharmacological strategies for the BBB treatment of neurological disorders, including stroke, neurodegenerative diseases, and brain inflammation. [31,32,33,39,65,66].

In conclusion, these data are the first to describe that PROK1 and PROK2 can modulate the permeability of the blood–brain barrier, thus potentially contributing to the regulation of cerebrovascular function and its potential relevance in various neurological disorders. In addition, novel effects of PROKs on neurons were revealed, such as PROK1-stimulated migration and the PROK2 inhibition of cell proliferation. However, further research is warranted to elucidate the underlying mechanisms and signaling cascades involved in prokineticin-mediated cellular responses. Understanding the functional significance of prokineticins in different cell types may provide valuable insights into their therapeutic potential in various physiological and pathological contexts, including angiogenesis, neuroprotection, and neurodevelopment. This knowledge may pave the way for the development of targeted therapeutic strategies to address BBB dysfunction in neurological disorders. A comprehensive understanding of such mechanisms may open new avenues for therapeutic intervention and improved patient outcomes in the fields of neurobiology and neurovascular research.

## 4. Materials and Methods

### 4.1. Compounds

Prokineticin 1 (PROK1, #ab177579) and prokineticin 2 (PROK2, #ab252913) were purchased from Abcam (Paris, France). Prokineticin receptor antagonist PC7 and PKRA antagonists: PC7 is a non-peptide PKOKR1-preferring antagonist that was obtained from Balboni [67]. PKR-A is a non-peptide PROKR2-preferring antagonist and it was obtained from Zhou [27]. 

### 4.2. Murine Brain Cell Culture

Cell lines were grown on plates of 75 cm^2^ in a final volume of 15 mL of media. We used murine brain microvascular endothelial cells (bEnd.3 [BEND3] (ATCC CRL-2299, LGC, Molsheim, France)), murine astrocytes (C8-D30 [Astrocyte type III clone] (ATCC CRL-2534, LGC, Molsheim, France)), DMEM high glucose (#41965-039 GIBCO, ThermoFisher Scientific, Waltham, MA, USA), and N2a murine Neuroblastoma (ATCC CCL-131) DMEM Glutamax (#31966-021 GIBCO, ThermoFisher Scientific). Mediums were supplemented with 10% fetal bovine serum (FBS, Eurobio Scientific, Les Ulis, France) and 1% penicillin-streptomycin (#15140-122 GIBCO, ThermoFisher Scientific) in addition to Non-Essential amino acids NEAA (#11140-050 GIBCO, ThermoFisher Scientific). Cells were maintained at 37 °C with 5% CO2. All cell lines were routinely tested for mycoplasma contamination using a MycoAlertTM PLUS kit (Lonza, Basel, Switzerland). Cell counting was performed using Trypan blue and read with a TC20™ Automated Cell Counter (Bio-Rad, Hercules, CA, USA).

### 4.3. Western Blot

A total of 5 × 10^7^ cells per cell line (bEnd.3, C8-D30, N2a), at 80% confluence, were extracted using RIPA lysis buffer (1% Triton X-1OO, 0.1% SDS, 150 mM NaCl, 50 mM Tris-base, 0.5% deoxycholatic acid, 0.5 mM EGTA, PH = 7.8) containing 0.1% protease inhibitor (Halt protease inhibitor, #78429 Thermo Scientific). After 30 min incubation on ice, using a cell scraper, the cellular lysates were recuperated and transferred into tubes for centrifugation (14,000 rpm) for 5 min at 4 °C, and the supernatants were collected. 

Protein concentrations were determined via BCA assay (using a Micro BCA protein Assay kit, #23235 Thermo Scientific). Proteins were denatured in Laemmli sample buffer (Laemmli, β-mercaptoethanol 5% (*v*/*v*), bomophenol blue) and boiled for 5 min. Proteins (30 μg) were loaded per lane and samples were electrophoretically separated on precast polyacrylamide gels (Mini-Protean^®^ TGX Stain-Free™ 4–20%). The proteins’ transfers were performed, using the rapid Biorad device (Trans-Blot turbo, program: mixed MW 7 min–25 V), onto 0.2 μm nitrocellulose membrane (Bio-Rad #1704271). For immunoblot analysis, the membranes were saturated using a blocking buffer [TBS (1X) Tween20 (0.1%) + 5% dry milk] for 1 h. Primary antibodies against PROKR1 (1:200, 0.2 μg/mL, Covalab, Bron, France) and PROKR2 (1:200, 0.53 μg/mL, Covalab, Bron, France) in incubation buffer [TBS (1X) Tween 20 (0.1%)] were added to the membranes and incubated overnight at 4 °C. β-actin β-actin clone AC-15 (1:1000, Sigma Aldrich, A5441, St. Louis, MO, USA) was used to confirm equal loading of the gels into each well. After three washes with TBS (1X)-Tween 20 0.1%, blots were incubated with HRP-coupled anti-rabbit or anti-mouse IgG (1:1000 in TBS (1X)-Tween 20 0.1%, Jackson ImmunoResearch, 115-035-062/111-035-144, West Grove, PA, USA) for 1 h at room temperature. Complexes were detected using the ECL Western blotting substrate system (#1705061, Bio-Rad) and chemiluminescence was detected using a iBright™ FL1500 Imaging System (#A4424, Invitrogen™, Waltham, MA, USA). Then, membranes were de-blotted using ReBlot Pus Mild Antibody Stripping Solution (#2502, Merck, Lowe, NJ, USA) to assess β-actin as loading control to normalize the total protein load in each lane. Quantification of the bands was conducted using Image J 1.53a software.

### 4.4. Proliferation Assay

A total of 6 × 10^3^ cells per cell line (bEnd.3, C8-D30, N2a) were seeded in 96-well plates and incubated (37 °C, 5% CO_2_) for the next day in presence of a specific medium for each cell line. The next day, cells were deprived by replacing the medium with one derived from FBS (1% FBS) for 5 h before adding ligands. PROK1 or PROK2 cells were incubated with or without PROK1 or PROK2 at different concentrations (0.5–30 nM) in serum-free media, for 40 h. A control group consisting of cells incubated without ligands was included. Proliferation was assessed using the IncuCyte^®^ Live Cell Analysis Imaging System (S3, Sartorius, France). The results are presented at the time point T = 40 h, for each given concentration of PROK1 or PROK2. All samples consisted of 3 replicates. Experiments were repeated at least 3 times. 

### 4.5. Wound-Healing Migration Assay

A total of 85,000 cells, per cell line, were seeded in 96-well plates (#4379, Corning, Corning, NJ, USA) and incubated (37 °C, 5% CO_2_) for the next day in presence of a specific medium for each cell line. The next day, when the cells reached 100% confluence, cells were deprived by replacing the medium with a medium with 1% FBS, for 5 h. Just before adding the ligands, the cells were scratched using the wound maker (Incucyte^®^ 96-well Woundmaker Tool, Essen Bioscience, Ann Arbor, MI, USA). Cells were then treated with increasing concentrations of PROK1 or PROK2 (0.5–30 nM) in serum-free media. Antagonists PC-7 or PKR-A were pre-incubated at 1 μM, 2 h before the ligands’ addition. Media was supplemented with Mitomycin C (1/1000, 0.5 mg/mL, Sigma Aldrich) to block cell proliferation. The follow-up of the wound closure was performed using the IncuCyte^®^ Live Cell Analysis Imaging System. Images were recorded every 2 h for up to 20 h, and analysis was performed using the Incucyte Scratch wound protocol (Essen Bioscience). A control group, consisting of cells without any PROKs treatment, served as a reference. All samples consisted of 3 replicates. Experiments were repeated at least 3 times.

### 4.6. Co-Culture of Endothelial Cells and Astrocytes

Contact and non-contact co-culture BBB models using immortalized brain capillary endothelial cells and immortalized astrocytes were established as follows. bEnd.3 (grown with initial medium: DMEM, 10% FBS, 1% penicillin/streptomycin) and C8D30 cells were seeded separately on the apical side of the filter PET membranes of 24-well plate culture inserts (6.4 mm diameter, with 3.0 μm pore; SABEU GmbH & Co. KG, Northeim, Germany), coated with rat-tail collagen type I (10 μg/cm^2^, #A10483-01, Gibco). The cell seeding density was 4 × 10^4^ cells/cm^2^ for bEnd.3. Beforehand, 4 × 10^4^ cells/cm^2^ of C8D30 cells were seeded on the rat-tail collagen type I coated (10 μg/cm^2^) on the basal side of the porous filter membrane and were allowed to attach for 3 h. The medium used for the co-culture was DMEM supplemented with growth factors (EGM™-2 MV Microvascular Endothelial Cell Growth Medium SingleQuots™; Lonza, Switzerland). The medium from both luminal and abluminal sides of the Transwell filter was changed every other day.

As nonconditioned controls, some of the bEnd.3-seeded filters were cultured in the absence of astrocytes. Also, collagen-coated inserts (without astrocytes and endothelial cells) were kept in the same conditions and used to estimate flux across the filter alone.

### 4.7. TEER Measurement

Under these conditions, the integrity of the bEnd.3/C8D30 model (hereafter called endo/astro model), the in vitro BBB model, was assessed by manually measuring the transendothelial electrical resistance (TEER) using the EVOM2 Epithelial Volt/Ohm Meter and an STX-2 electrode system (World Precision Instruments LLC, Sarasota, FL, USA) for 6–7 days. TEER values for cell layers, expressed in Ω.cm^2^, were calculated by subtracting the resistance of a coated control insert without cells from a coated insert with cells and by subsequent correction for surface area. Subsequently, the experiments were performed when TEER values reached a plateau (i.e., the day of maximum barrier integrity, usually 7 days after seeding). Results are expressed as means ± S.E.M, and represent 3–5 independent experiments constituting of 2–3 replicates for each experiment.

### 4.8. Permeability of FITC-Dextran

The endothelial barrier integrity was evaluated by measuring FITC-dextran permeation on the inserts. The prokineticin effect on BBB integrity was tested via incubation of ligands directly or after 1 h preincubation with or without the receptors’ antagonists, PC7 and PKR-A. After overnight treatments, the permeability of the barrier was evaluated regarding the apical-to-basolateral transport of fluorescein isothiocyanate-conjugated dextran (FD-40, MW 40,000; Sigma-Aldrich) [47]. FD-40 (1 mg/mL) in HBSS Media (Gibco, Grand Island, NY, USA) was added to the apical compartment and the media in the basolateral compartment was replaced with HBSS. After 60 min at 37 °C in a 5% CO_2_ incubator, the samples collected from the apical and basolateral compartments were moved onto a 96-well plate. The fluorescence of the samples (I.F.U) was measured on a fluorescence luminometer (Tecan Systems Inc., Seestrasse, Mannedorf, Switzerland) at the excitation wavelength of 485 nm and emission wavelength of 535 nm. To determine the permeability of FITC-dextran across the BBB co-culture cells, the slope of the clearance curves (PS = permeability × surface area product; in µL·min^−1^) corresponding to the volume cleared from the apical to the basolateral compartment (in µL) for 60 min was calculated for the control filters without cells and for those with bEnd.3 and C8D30 cells, denoted as PSf and PSt, respectively. From these values, the PS values for BLECs (PSe) were determined according to the formula “1/PSe = 1/PSt + 1/PSf”. Then, the Pe values for the insert with BBB were obtained using the calculation “Pe = PSe/S”, with PSe in cm^3^·min^−1^ and S being the filter surface area (0.33 cm^2^). To determine the effect of PROK1 and PROK2 on FD-40 passage across the BBB, we compared the percentage of the applied tracer in the basolateral insert across the co-culture cell to that across cell-free inserts. Transport of FD-40 across the cell-free inserts was used as a measure of spontaneous passage of the tracer across the co-culture and was reported as % of spontaneous passage and measure of the BBB integrity. The data are shown as means ± S.E.M of 3–5 independent biological repeats.

### 4.9. Statistical Analysis

Analyses were conducted using GraphPad Prism version 9.5.0 (GraphPad Software, Inc., La Jolla, CA, USA). Data are presented as means ± SEM. Comparisons between 2 groups were statistically evaluated using unpaired two-tailed *t*-tests. Multiple comparisons were statistically evaluated via a one-way or 2-way ANOVA with Sidak post-test. *p* values less than 0.05 were considered significant.

## Data Availability

The data presented in this study are available on request from the corresponding author.
